# Improving the Management of Post-Operative Hypocalcaemia in Thyroid Surgery

**DOI:** 10.7759/cureus.15137

**Published:** 2021-05-20

**Authors:** Rachael Collins, George Lafford, Rebecca Ferris, Jeremy Turner, Peter Tassone

**Affiliations:** 1 Otolaryngology, Norfolk and Norwich University Hospital NHS Foundation Trust, Norwich, GBR; 2 Plastic Surgery, Norfolk and Norwich University Hospital NHS Foundation Trust, Norwich, GBR; 3 Endocrinology, Diabetes and Metabolism, Norfolk and Norwich University Hospital NHS Foundation Trust, Norwich, GBR; 4 Otolaryngology - Head and Neck Surgery, Norfolk and Norwich University Hospital NHS Foundation Trust, Norwich, GBR

**Keywords:** quality improvement projects, otolaryngology-head & neck surgeons, otolaryngology education, postoperative hypocalcemia, quality improvement and patient safety, thyroid supplementation

## Abstract

Hypocalcaemia is a frequent, and potentially dangerous complication of total thyroidectomy occurring secondary to devascularisation of the parathyroid glands. This quality improvement (QI) project was undertaken in a large Ear, Nose and Throat (ENT) department in the East of England over a one year period. The project aimed to improve postoperative guideline compliance by optimising the recognition and management of patients at risk of hypocalcaemia. This process focussed on improving parathyroid hormone (PTH) and calcium blood testing, prophylactic calcium prescribing and the subsequent monitoring and management of hypocalcaemia.

A baseline audit was conducted to determine the initial guideline compliance. The QI process subsequently involved the introduction of a new intraoperative PTH pathway and the amendment of trust guidelines. In addition, there was a focus on improving clinician awareness of guidelines, junior doctor education, communication between operating surgeons and junior doctors and the optimisation of patient handover.

The project saw a significant improvement in the monitoring of hypocalcaemia (from 22.2% to 83.3% for patients with an intermediate risk of hypocalcaemia) and in the prescribing of prophylactic calcium supplements from 7.5% to 43.5%. The measurement of PTH at four hours improved from 42.5% to 52.2%. By optimising postoperative care, this QI project improved patient safety as well as impacting on the duration, and overall cost, of inpatient stay.

## Introduction

Hypocalcaemia is a frequent, and potentially the most dangerous complication of total thyroidectomy [1,2]. At the Norfolk and Norwich University hospital (NNUH), thyroidectomy patients are managed as inpatients overnight and, if there are no complications, the patients are discharged 24 hours later. The NNUH is part of an NHS Foundation Trust serving a large geographic area with patients referred from throughout Norfolk and north Suffolk. The Ear, Nose and Throat (ENT) department consists of eight consultants, seven registrars, and seven senior house officers, performing in the region of 60 total, or completion, thyroidectomies a year.

During the perioperative period, patients have two sets of blood tests, at 4-hours and day 1 (12-hours) post operation. The first blood test consists of both calcium levels and parathyroid hormone (PTH). The second blood test measures calcium levels alone. The measurement of PTH levels post-thyroidectomy allows for early risk assessment of hypocalcaemia [3] and are an integral part of local guidelines in identifying and appropriately managing affected patients. In addition, local guidelines state that patients should be prescribed prophylactic calcium supplements. For those patients deemed to be at intermediate or high risk of developing hypocalcaemia (based on the PTH value), local guidelines advise on medical treatment and timing of further blood tests, namely repeated calcium monitoring at 12-hourly intervals and titrating up of calcium supplements [4].

Within the department, it was noted that PTH levels were often not available within the appropriate timeframe and there were challenges in identifying and treating patients who developed hypocalcaemia. It was also noticed that there was some variation in practice within the department regarding prophylactic prescribing of calcium supplements.

This quality improvement (QI) project identified several contributing factors including the pathway for the laboratory processing of PTH levels, communication between senior and junior colleagues, education and awareness of guidelines among junior doctors and the design of local guidelines. Over the course of a year, the project aimed to ensure 100% of patients who underwent total or completion thyroidectomies had the appropriate PTH and calcium levels taken during the postoperative period, that all were prescribed anticipatory calcium supplements and, for those patients identified at risk of hypocalcaemia, the appropriate frequency and duration of calcium monitoring was undertaken during the postoperative period.

Hypocalcaemia has been reported to occur in 24.9% of post-thyroidectomy patients on day 1 post-op [5]. Hypocalcaemia can range in its severity from mild symptoms such as muscle spasms and cramps to serious and life-threatening sequelae including seizures, personality disturbances and prolonged QT intervals [6]. It is therefore essential that the condition is detected and treated appropriately in the postoperative period. Individual trusts develop their own local clinical guidelines in line with the British Association of Endocrine and Thyroid Surgeons (BAETS) recommendations [4].

BAETS guidelines suggest calcium should be checked at 12 hours post operation and, on the basis of the result, advise on medical treatment and timing of further blood tests, however, they encourage the creation of local guidelines. Local guidelines were configured to incorporate PTH levels into the postoperative management of total, or completion, thyroidectomy patients [4]. This allows for earlier detection of patients at risk of hypocalcaemia as well as facilitating quicker discharge for patients deemed to be at low risk. BAETS offer no specific guidance on administering prophylactic calcium and vitamin D supplements to this patient group, however, there is evidence that this can reduce postoperative hypocalcaemia and some trusts recommend their use [7,8].

Trusts vary in their approach in the post-operative management of total, or completion, thyroidectomy patients. The Royal Devon and Exeter Hospital manage their patients in the outpatient setting from day 1 post-operation [9]. Having identified a similar challenge in managing this patient group appropriately, they conducted a QI project focussed on patient information leaflets and optimising outpatient follow-up. Part of this project also involved improving clinician awareness and access to guidelines. Overall guideline compliance was increased from 79% to 100% [9]. An audit from a trust in West Yorkshire, which developed similar guidelines to those of the NNUH, identified a similar challenge with the attainment of post-operative blood. This audit found that not all patients had the specified calcium and PTH levels taken within four hours at 37.7% and 26.7%, respectively [10]. This audit highlighted inter-surgeon variation in practice as well as out of hour junior colleagues as contributing to this problem [10].

## Materials and methods

Data collection initially focussed on all patients who underwent a total or completion thyroidectomy over a six-month period between September 2018 and February 2019, this included 40 patients in total. Patient data was collected from the hospital's information Services, online biochemistry reports, and prescribing records from Electronic Prescribing Medicines Administration (EPMA). The following information was recorded: operation date, date of birth, surgeon, specialty, operation type, whether PTH and calcium were measured 4-hours post-op along with values, whether calcium was measured the next day along with values, whether the patient was hypocalcaemic, and whether they were prescribed the appropriate medication. All patients undergoing surgery had baseline bloods taken preoperatively including calcium levels. 

This local retrospective audit found the rate of hypocalcaemia, using the definition of adjusted calcium <2.1 mmol/L, to be 18.42% (n =7), which is lower than the national average [5]. The audit revealed that less than half (42.5%, n=17) had PTH levels taken within the 4-hour target post operation. In addition, only 7.5% (n=3) of patients were prescribed prophylactic calcium supplements. Of the nine patients identified at intermediate risk of developing hypocalcaemia, 22.2% (n=2) received the appropriate 12 hourly calcium monitoring over a 48-hour period. There was only one patient during this time who was deemed at high risk of hypocalcaemia, and they were not monitored for 48 hours. This initial audit highlighted challenges in both identifying patients at risk of developing hypocalcaemia and in the initial management of hypocalcaemia. This audit cycle was repeated to evaluate the attainment of the project's aims: namely increasing the rate of biochemistry tests, prophylactic prescribing, and the monitoring of those at risk of developing hypocalcaemia.

**Figure 1:**
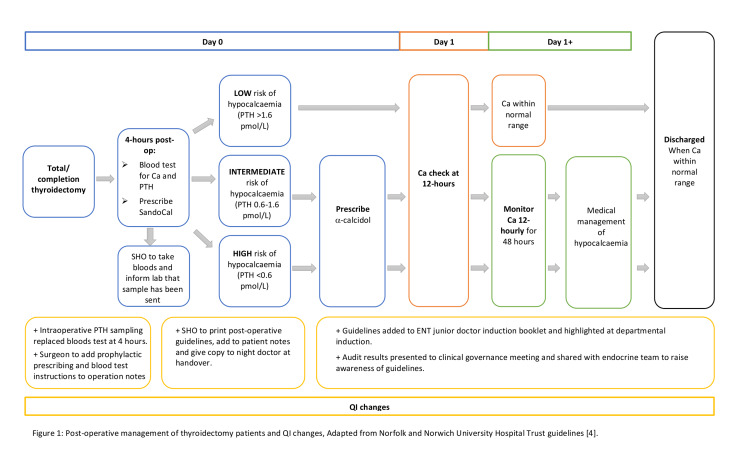
Post-operative management of thyroidectomy patients and QI changes; adapted from the Norfolk and Norwich University Hospital Trust guidelines QI: quality improvement; PTH: parathyroid hormone.

The QI team consisted of a head and neck consultant and two junior doctors. Stakeholder involvement was encouraged through presentation of the initial audit results at a regional governance meeting and a meeting with biochemistry professionals and endocrine specialists who were involved in the laboratory processing of blood samples, and in the creation of the original hospital guidelines, respectively. This helped the QI team to identify both areas for improvement and implement change. To encourage stakeholder involvement and engagement, these meetings were repeated as the ‘Plan, Do, Study, Act’ (PDSA) cycle evolved. The process of post-operative management of thyroidectomy patients and the QI plan is summarised in Figure 1. It represents the post-operative pathway for patients undergoing thyroid surgery. 

The main areas highlighted for QI

1. Pathway for PTH requesting. As the PTH level falls within minutes of devascularisation of the parathyroid glands, the PTH sample can be taken intraoperatively [11]. A new pathway for requesting PTH was created, this included the creation of a specific request section on the Integrated Clinical Environment (ICE) system and amendment of trust guidelines. This eliminated the requirement of the Senior House Officer (SHO) to take post-operative bloods or phone the laboratory to inform them.

2. Prescribing. Operating surgeons changed their practice by incorporating prescribing instructions into the operation notes. These instructions were incorporated into the handover document by SHOs.

3. Management of hypocalcaemia. SHOs from the day team printed copies of the post-operative guidelines adding them to the patients' notes as well as giving a copy to the night SHO at handover

4. Junior doctor education. Junior doctor awareness of the guidelines was increased through the introduction of an ENT SHO handbook, inclusion of the guidelines at departmental induction, and by displaying the guidelines in the ENT doctor’s office.

The QI team implemented most changes over the course of a month, in March 2019, with the involvement of the ENT colleagues, endocrine specialists, and biochemistry professionals. The QI process continued beyond this, over the course of the subsequent six months, as the PDSA cycles evolved.

Pathway for PTH requesting

PDSA cycle 1: The delay in postoperative PTH sampling was felt to be due to the limited time available for the laboratory to process the blood sample in time for the morning ward round the following day. The initial intervention focussed on increasing this time by ensuring a blood sample reached the laboratory earlier in the day. The decision was taken to change practice by taking PTH sample in theatres, within 30 minutes of removal of the thyroid tissue. During an interdepartmental meeting involving senior endocrine clinicians, ENT consultants, and laboratory managers, it was agreed that trust guidelines would be amended to adopt this new practice. The aim was to drastically increase the percentage of patients who had a PTH result in time for day 1 postoperative review.

PDSA cycle 2: The laboratory receives numerous PTH samples from throughout the hospital. Biochemistry staff highlighted that they were not always able to easily identify the thyroidectomy patient’s samples. Previously the SHO would phone the laboratory to highlight these samples for processing, however, this was often overlooked. To eliminate this, a new blood request option labelled ‘intraoperative PTH’ was added to ICE, which automatically highlighted these samples to the laboratory.

PDSA cycle 3: It was recognised that not all staff were aware of the new process for PTH sampling of thyroidectomy patients. The changes were therefore highlighted at a local departmental governance meeting. In addition, an email with the new guidelines was circulated to operating surgeons.

Prescribing

Operating surgeons would routinely provide post-operative plans in the operation notes of each patient including instructions for the timing of blood tests. However, specific prescribing instructions were generally not included. It was felt that including instructions for the routine prescribing of prophylactic calcium supplementation would improve prescribing rates as these plans were routinely checked by SHOs and added to the patient handover document. This was discussed in a departmental governance meeting and surgeons adopted this practice.

Management of hypocalcaemia

PDSA cycle 1: It was felt that improving awareness of the trust guidelines on postoperative management of hypocalcameia, particularly among the junior doctors, was key to improving patient identification and management. The aim was to ensure that all patients deemed at intermediate or high risk of hypocalcaemia as per their PTH result (Figure 1) would have the appropriate serum calcium monitoring. In the first instance, awareness was increased by highlighting the baseline audit findings and the postoperative guidelines at a departmental governance meeting.

PDSA cycle 2: Subsequently, for patients at risk of developing hypocalcaemia, a copy of the trust guidelines was printed and inserted into the patients' notes on the ward. This further highlighted the guidelines to all members of the team, including nursing staff, as well as making them easily accessible.

PDSA cycle 3: It was felt that the guidelines were particularly challenging for non-departmental SHOs overnight. The ENT SHOs adopted the practice of giving a copy of the guidelines to the night SHO at handover, highlighting their calcium results and any intervention they may require overnight. This removed the need for the night SHO to locate or interpret an unfamiliar guideline overnight.

Junior doctor education

It was thought that through the education of junior doctors, particularly ENT SHOs, there would be an increased awareness and understanding of the guidelines, and a collective responsibility to follow them. This would improve prophylactic calcium prescribing and the identification and management of patients at risk of hypocalcaemia. An ENT SHO induction handbook was updated to incorporate the guidelines, this was emailed to new SHOs before starting in the department. The guidelines were also highlighted to new doctors at departmental induction. In addition, a flowchart of the guideline was displayed in the ENT doctor’s office.

## Results

To evaluate the impact of the QI project, a re-audit was conducted from end of March to October 2019, using the same parameters as the baseline audit. This included a total of 23 patients. The rate of hypocalcaemia, using the definition of adjusted calcium <2.1 mmol/L, fell following the QI project from 18.4% to 13.1%.

The measuring of PTH at 4 hours post-operatively improved from 42.5% to 52.2%. Unfortunately, the percentage of patients having a PTH sample within 24 hours of surgery has fallen slightly from 90.0% of patients to 87.0%.

The monitoring of hypocalcaemia improved significantly; 83.3% (n=5) of patients who were at an intermediate risk of hypocalcaemia (PTH 0.6-1.6) had 12 hourly calcium monitoring for 48 hours compared to 22.2% prior to the QI project. In the re-audit, two patients were identified to be at high risk of hypocalcaemia (PTH <0.6). They both had 12-hourly Ca measurement for 48 hours compared to 0% in the previous audit.

Although the overall rate and monitoring of hypocalcaemia improved, prescribing as per the guidelines worsened. Of those that had mild hypocalcaemia (n=3), only 33.3% were prescribed sandocal 1000 iii BD and 33.3% 1α-calcidol compared to 71.4% and 42.9% prior to the QI project, respectively. Although it must be noted that the baseline audit had over double the number of patients with hypocalcaemia (n=7) compared to the re-audit. Prophylactic prescribing of sandocal 1000 ii BD improved significantly from 7.5% to 43.5%.

## Discussion

Lessons and limitations

The project’s aim was to improve PTH and calcium monitoring in the postoperative period for patients who underwent complete thyroidectomies, as well as improve calcium supplement prescribing. The main gains from this QI project were significantly increasing both the prophylactic prescribing of calcium supplementation for all patients and the monitoring of patients who were hypocalcaemic postoperatively.

It is encouraging that the overall rate of hypocalcaemia fell following the QI project and, while other factors beyond the project may contribute to this, it is reasonable to assert that increased rates of prophylactic calcium prescribing may be partly responsible. This resulted in improved patient satisfaction, shorter hospital stay, and reduced costs. The intervention that proved the most effective here was the addition of prescribing instructions in operation notes; this demonstrates how a simple change in practice can result in significant gains.

Improving clinical awareness and education was an area of focus of this project. During the QI process, it became apparent that clinicians were not always aware of the guideline advice, especially amongst junior colleagues. The PDSA cycles were particularly important here as different areas were highlighted and targeted over the space of six months from new SHO induction to the evening patient handover.

While the rates of initial measurement of PTH (within four hours) improved, the QI project did not result in the substantial gain hoped for. Prior to the implementation of the intraoperative PTH pathway, the project encouraged interdepartmental involvement and engagement through a series of meetings and the amendment of the trust's guidelines. Nevertheless, it can be challenging for long-term staff to embrace new practice and success may have been limited by staff buy in. While this pathway was generally adopted, the time to process the PTH sample remained limited by the working hours of the laboratory. The intraoperative PTH pathway remains part of the trust's guidelines and work is ongoing to increase the processing time of PTH samples.

Unfortunately, for the patients who had mild hypocalcaemia, the prescribing rates of calcium replacement, as per the guidelines, decreased. However, only three patients in the repeat audit were found to have mild hypocalcaemia and so it is difficult to infer the impact of the QI project in this area. It is interesting that while monitoring of calcium levels has improved acting on the results did not. Further quality improvement work is required to optimise the medical management of patients with hypocalcaemia.

Despite having a large ENT department at the NNUH, and auditing patients over a year, the sample size of this project was limited by the number of operations performed. This restricted the ability of the QI team to re-measure throughout the improvement process. In addition, the re-audit had lower numbers of patients (n=23) compared to the baseline audit (n=40). This may be partly due to the time of year the re-audit took place, which included the summer holiday season. This period also included the beginning of August where all SHOs rotate specialties. While the project aimed to improve the induction of new SHOs, this changeover may have negatively impacted some of the QI outcomes. 

The sustainability of interventions is affected by the regular changeover of staff. Raising the changes described during the induction of new SHOs aims to address this. 

## Conclusions

Improving the recognition and management of postoperative hypocalcaemia in thyroid surgery is important not only for patient safety but also for patient satisfaction, the duration of inpatient stay, and overall cost of care. This QI project has identified a range of factors that contribute to how effective this process is including the laboratory processing of blood samples, clinician education and awareness of guidelines, and patient handover. The QI process identified similar factors that contribute to guideline compliance as previous projects in this area including clinician awareness, patient handover, and the importance of encouraging clinician involvement. In addition, this project evaluates the introduction of a new pathway for PTH processing and the challenges associated with it. While it may be initially disappointing, more substantial gains were not achieved in attaining earlier PTH samples the pathway is in its early days and continues to become established within the department. Significant gains were made in prophylactic prescribing and it is encouraging that a small change in practice can improve outcomes. Further QI work is needed to impact prescribing for hypercalcaemic patients.
